# Translation of *Fit & Strong!* for Middle-Aged and Older Adults: Examining Implementation and Effectiveness of a Lay-Led Model in Central Texas

**DOI:** 10.3389/fpubh.2014.00187

**Published:** 2015-04-27

**Authors:** Marcia G. Ory, Shinduk Lee, Alyson Zollinger, Kiran Bhurtyal, Luohua Jiang, Matthew Lee Smith

**Affiliations:** ^1^Department of Health Promotion and Community Health Sciences, Texas A&M Health Science Center, School of Public Health, College Station, TX, USA; ^2^Office of Surveillance, Evaluation, and Research, Texas Department of State Health Services, Austin, TX, USA; ^3^Department of Epidemiology and Biostatistics, Texas A&M Health Science Center, School of Public Health, College Station, TX, USA; ^4^Department of Health Promotion and Behavior, College of Public Health, The University of Georgia, Athens, GA, USA

**Keywords:** evidence-based program, aging, exercise, implementation research

## Abstract

The *Fit & Strong!* program is an evidence-based, multi-component program promoting physical activity among older adults, particularly those suffering from lower-extremity osteoarthritis. The primary purpose of the study is to examine if the *Fit & Strong!* program translated into a lay-leader model can produce comparable outcomes to the original program taught by physical therapists and/or certified exercise instructors. A single-group, pre–post study design was employed, and data were collected at the baseline (*n* = 136 participants) and the intervention conclusion (*n* = 71) with both baseline and post-intervention data. The measurements included socio-demographic information, health- and behavior-related information, and health-related quality of life. Various statistical tests were used for the program impact analysis and examination of the association between participant characteristics and program completion. As in the original study, there were statistically significant (*p* < 0.05) improvements in self-efficacy for exercise, aerobic capacity, joint stiffness, level of energy, and amount and intensity of physical activities. The odds of completing the program were significantly lower for the participants from rural areas and those having multiple chronic conditions. Successful adaptation of the *Fit & Strong!* program to a lay-leader model can increase the likelihood of program dissemination by broadening the selection pool of instructors and, hence, reducing the potential issue of resource limitation. However, high program attrition rates (54.1%) emphasize the importance of adopting evidence-based strategies for improving the retention of the participants from rural areas and those with multiple chronic conditions.

## Introduction

In recent years, there has been growing interest in evidence-based disease prevention programs that help middle-aged and older adults improve their health and quality of life through self-management strategies. This greater attention has resulted, in part, from an emerging recognition that adults of all ages including older adults can benefit from health promotion programs (1, 2) and a larger appreciation of the value of evidence-based approaches (3–5). Many of these programs incorporate elements to increase physical activity among participants (6–8) because of the importance of mobility for sustained independent living (9, 10). *Fit & Strong!* is an example of one such multi-component physical activity program that combines guided aerobic, strength, and flexibility training with health education (7, 11). Previously tested in a randomized clinical trial, *Fit & Strong!* has demonstrated efficacy to improve participants’: (1) self-efficacy (SE), or confidence, for exercise; (2) physical activity adherence; (3) aerobic capacity; and (4) lower-extremity joint pain and stiffness (7, 11).

After a series of successful efficacy trials, the program developers have proactively partnered with multiple agencies to disseminate *Fit & Strong!* to more diverse populations and settings (12). While originally developed for older adults with lower-extremity osteoarthritis, it is now being marketed more broadly as an evidence-based physical activity/behavioral change program that can be delivered to sedentary, older adults through aging services, and public health networks (13, 14).

Despite the potential advantages of widely disseminating *Fit & Strong!* in community settings, some challenges were anticipated in the actual delivery through the aging services network in Central Texas. One identified translational research problem was resource limitation related to the inadequate availability of trained instructors (15, 16). Prior to its translation in Central Texas, eligible instructors for *Fit & Strong!* were limited to physical therapists (PTs) and certified exercise instructors (CEIs) as a means to ensure safety and effectiveness in conducting the program (7, 11, 17). However, this narrow pool of eligible instructors limits possibilities for grand-scale uptake and dissemination. Therefore, in collaboration with the program developers, efforts were taken to modify the instructor criteria and expand the types of instructors deemed appropriate to deliver the *Fit & Strong!* program.

Given the growth of other evidence-based programs delivered using train-the-trainer, lay-leader models through a variety of aging, public health, and health care organizations, questions arose about whether *Fit & Strong!* might similarly be translated to a lay-led model and, thus, broaden the selection pool of instructors and minimize the anticipated resource limitation problem. Of primary concern was whether instructors trained in other evidence-based programs, without exercise training experience, could safely deliver *Fit & Strong!* to seniors with non-specific chronic conditions while maintaining program effectiveness. In response to such questions, this study examined the adaptation of *Fit & Strong!* to a lay-leader model in Central Texas using a quasi-experimental study design. The specific purposes of this study were to: (1) describe the characteristics of participants enrolled in the translated *Fit & Strong!* program; (2) examine factors associated with program attendance; and (3) assess changes in health-related outcomes among participants.

## Materials and Methods

### *Fit & Strong!* intervention

The *Fit & Strong!* program is a multi-component physical activity and behavioral change program that is structured around two key components: (1) participation in group-facilitated (or guided) physical activity; and (2) group-based health education/problem-solving. Over an 8-week period, individuals participate in 24 total sessions, meeting 3 days each week for 90-min each session. Each session begins with 60-min of structured physical activity, which is then followed by a 30-min group-based discussion/problem-solving period (7, 11, 17).

The physical activity component includes: (1) warm-up exercises (5–10 min); (2) low-impact aerobic conditioning (e.g., walking and step aerobics) where participants begin with 10 min of activity and gradually work up to 30 min by the end of the program; (3) strength exercises (primarily lower-extremity) using ankle weights and resistance bands (15–20 min); and (4) cool-down and flexibility exercises (5–10 min) (7, 11, 17). During the group-based discussion/problem-solving component, instructors lead participants in discussions of various health-related topics relying on a program curriculum guide. These interactive sessions are intended to help participants make healthy changes that sustain long-term healthy lifestyle management (e.g., improved arthritis symptom management and physical activity engagement). Toward the end of the program, participants are also encouraged to create an individualized physical activity plan to enable and promote continued physical activity after the 8-week program concludes (7, 11, 17).

### Adaptation of the *Fit & Strong!* program

The proposed adaptation of *Fit & Strong!* in Central Texas involved two modifications: (1) a shift in the required instructor qualification from exercise-experts (i.e., PTs or CEIs) to lay-leaders; and (2) a modification in the training protocol. In response to a shortage of qualified instructors in the targeted communities, especially in the rural sites, program implementers at the Texas A&M Program on Healthy Aging collaborated with the original program developers at the University of Illinois – Chicago to modify the existing qualification requirement for instructors and allow lay individuals to lead *Fit & Strong!* classes. For lay individuals to be qualified to lead *Fit & Strong!* classes, the lay-leaders needed to meet the following criteria: (1) be certified in another evidence-based class (e.g., *A Matter of Balance, Chronic Disease Self-Management Program*, or *Diabetes Self-Management Program*) and have experience and comfort leading group classes, if not already a PT or a CEI; (2) participate in the full instructor and supplemental lay-leader trainings conducted by the Master Trainers; and (3) adhere to fidelity standards by following the training manual in conducting the program (18, 19).

Recruitment efforts for lay-leaders consisted of collaboration with community stakeholders who were instrumental in: (1) referring and identifying qualified/capable individuals; and (2) disseminating information (e.g., flyers and emails) about the lay-leader training.

Training for the original *Fit & Strong!* program was conducted by *Fit & Strong!* staff, Master, and T-Trainers (e.g., the most experienced trainers who are able to train and certify Master trainers). Lay individuals as well as CEIs completed the mandatory instructor training, which lasted 8 h (in 1 day) and covered: (1) program background & development; (2) importance of fidelity; (3) roles/responsibilities of instructors in relation to other *Fit & Strong!* team members (e.g., developers); (4) *Fit & Strong!* exercise components (description and demonstration of various types of exercises used throughout the program); (5) *Fit & Strong!* group discussion/problem-solving component (including role plays, facilitator management roles); and (6) data collection, evaluation, and fidelity responsibilities of instructors (18, 19). Lay individuals then completed an additional day of training (half-day, 4 h) tailored to lay-leaders that emphasized basic exercise principles and safety as they applied to the *Fit & Strong!* program.

### Implementation and fidelity of the translated *Fit & Strong!* program

During the implementation of the adapted *Fit & Strong!* program in Central Texas, the Texas A&M *Fit & Strong!* evaluation team along with program developers engaged in best practice quality assurance strategies to assure that the adapted program would be delivered with fidelity (20). This included: (1) fidelity assessments (using a specified fidelity checklist) through observations at the delivery sites; (2) setting up a mechanism for frequent communication with the lay-leaders; and (3) conducting process evaluations of program implementation and participant experiences. The program evaluations assessed participants in terms of: (a) attendance; (b) experiences with the program and instructors; and (c) program impact. Evaluations also included instructors’ experiences and assessment of the program (instructor manual, group discussion, and exercise components) as well as the effectiveness of the instructor training. The fidelity assessments and program evaluations provided further guidance and support for instructors in conducting classes more effectively and correctly. These quality assurance strategies helped reinforce adherence to the curricula material presented through the original program manuals.

### Program setting and delivery

Five intervention sites were selected from Central Texas, and 12 different *Fit & Strong!* classes were offered across the various sites from September 2012 through June 2013. Site selection was based on three interrelated criteria: (1) community support for hosting *Fit & Strong!* classes (community “buy-in” was seen as a critical factor for both recruitment and sustainability), (2) facility availability for *Fit & Strong!* classes, and (3) the presence of a sufficient number of older adults who could benefit from *Fit & Strong!* and who were interested in participating in the program. The number of participants in each class ranged from 16 to 25, which roughly paralleled the recommended 20–25 participant maximum ideal (21). Institutional review board approval was obtained at Texas A&M University.

Local senior centers, community centers, and health resource centers served as host agencies for the delivery of the *Fit & Strong!* classes. These host agencies also assisted with program promotion and participant recruitment. For example, a couple of agencies hosted promotional meetings for the program as well as voluntarily conducted on-site program enrollment while coordinating these efforts with Texas A&M program implementers. Many of these agencies expressed appreciation for the offering of a new program at their sites and, thus, were more willing to volunteer their services to assist with promotion and recruitment endeavors.

### Participants

Study participants included adults aged 47–94 years who enrolled in *Fit & Strong!* in rural and urban counties in Central Texas between 2012 and 2013. All middle-aged and older adults residing in the area were eligible to enroll in the program; however, only those who had never previously participated in a *Fit & Strong!* class and attended the first or second class session and also completed a baseline survey were included in the study analyses (*n* = 136). As previously mentioned, participants were recruited by host agency members as well as Texas A&M program implementers. Participants were recruited through various sources, including print materials (e.g., program guides, brochures/flyers, and newspaper postings), community resources (e.g., senior clubs/classes and promotional meetings), family or friends (word of mouth), and health care providers. The majority of participants were recruited through print materials (43.4%) and family or friends (28.7%).

### Measures

Data sources included a baseline survey at the beginning (first and second sessions of each class), a post-test survey at the end of the 8-week program (final week), and an attendance log. Demographic data that was drawn from the baseline survey included age, gender, race/ethnicity, education, marital status, employment status, and annual household income. Several outcome measures were extracted and analyzed based upon these baseline and post-test surveys. Primary outcomes included: (1) SE for exercise and (2) level of physical activity related to aerobic capacity, flexibility, and strength. Secondary outcomes included: (1) self-rated health status; (2) joint pain and stiffness; and (3) level of energy (fatigue). Paralleling assessment protocols being utilized by the original program developers in their program dissemination phase (22), the measurement battery was designed to be administered to older adults in community settings. The surveys were designed to be completed on average in <20 min. Program staff was available during data collection to assist older adults when filling out the forms, as needed.

#### Self-efficacy for exercise

Self-efficacy for exercise was measured using four items. The items asked how confident participants are in performing different types of exercise (e.g., strength and flexibility), performing vigorous exercises, and performing exercise despite pain or symptoms. Each item is based on a 10-point scale ranging from “not at all confident” (score = 1) to “totally confident” (score = 10). The score for SE for exercise was the mean of the four items. Higher SE scores indicated *higher self-efficacy*. The scale value was set to “missing” if more than one item was missing (23); based on the criteria, seven total *missing* cases were omitted from the analyses. If only one item was missing, the mean of the remaining three items was used. Internal reliability was high for this composite scale (Cronbach’s α = 0.96).

#### Aerobic capacity, flexibility, and strength

A slight adaptation of the rapid assessment of physical activity (RAPA) was used to measure the amount and intensity of participants’ physical activity (24). The adapted RAPA consisted of eight items, and each item had a “yes” and “no” option. The first six items, which measured the intensity and frequency of physical activity were used to assess *aerobic capacity*.

The six items were: (1) I rarely or never do any physical activity; (2) I do some light or moderate physical activities but not every week; (3) I do some light physical activity every week; (4) I do moderate physical activity every week; (5) I do 30 min or more per day of moderate physical activity, five or more days per week; and (6) I do 20 min or more per day of vigorous physical activities, three or more days per week. Each of the six items reflected a specific level of aerobic capacity. For example, affirmative response to the item “(1)” represents “*sedentary*” and was scored 1; affirmative response to the item “(2)” represents “*under-active*” and was scored 2; affirmative response to the item “(3)” represents “*under-active regular – light activities*” and was scored 3; affirmative response to the item “(4)” represents “*under-active regular*” and was scored 4; and affirmative response to items “(5)” and/or “(6)” represents “*active*” and was scored 5. The highest score among the six items was selected for the aerobic capacity score (25). The remaining two items assessed *strength* and *flexibility*, and affirmative response to each item was scored 1. The strength and flexibility items were summed for descriptive purposes. The summed scale ranged from 0 to 2 (0 = none, 1 = either, and 2 = both).

#### Self-rated health

A single item was used to assess self-rated health (26), which has been identified as an outstanding predictor of future health (27). This item was a five-point scale with lower values indicating worse *health* (poor = 1) and higher values indicating *better health* (excellent = 5).

#### Joint pain and stiffness

The Western Ontario and McMasters University Osteoarthritis Index (WOMAC) was used to measure lower-extremity pain and stiffness (28). The adopted WOMAC consisted of seven items: five pain and two stiffness items. All seven items were in a five-point Likert scale structure ranging from “none” (score = 0) to “extreme” (score = 4). Scores for each section were summed to produce composite scales for pain and stiffness. The pain-scale ranged from 0 to 20 with higher values indicating *greater pain*; and the stiffness-scale ranged from 0 to 8 with higher values indicating *greater stiffness*. Internal reliabilities were high for both composite scales (Cronbach’s α = 0.89 for pain; 0.86 for stiffness) (29).

#### Level of energy and fatigue

The level of energy and fatigue was measured using five items (30). Each item was a six-point scale ranging from “none of the time” (score = 0) to “all of the time” (score = 5). Some scores were recoded to have an equal direction of answers among the five items (i.e., higher scores indicate *worse health*). The mean of the five items was used as the composite scale for the level of energy and fatigue. The scale ranged from 0 to 5 with higher values indicating a *lower level of energy* and a *greater level of fatigue*. Internal reliability was high for this composite scale (Cronbach’s α = 0.90).

#### Successful class completion

Attendance was tracked via attendance logs for each session, and the attendance data were used to calculate the attendance and completion rates. “Completion” was defined as attending at least 18 out of the 24 total *Fit & Strong!* sessions per class offering.

### Recruitment flow

**Figure 1:**
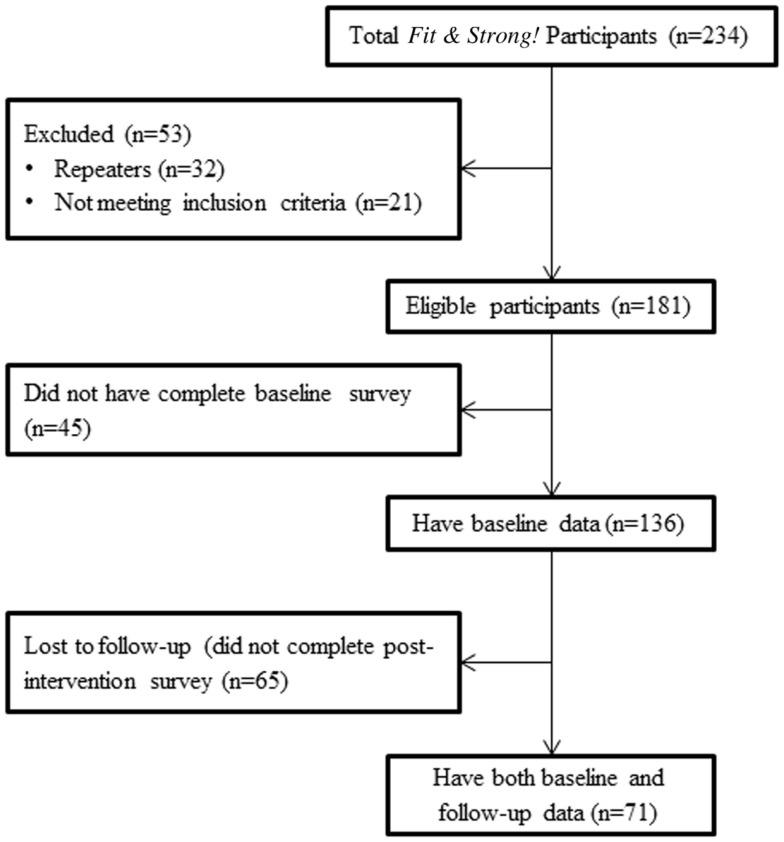
**Recruitment flow diagram for *Fit & Strong!* participants**.

The recruitment flow from initial program enrollment is presented in Figure 1 as a consort type diagram. This figure begins with all “participant enrollees” and concludes with eligible participants with linked baseline and post-test data who were treated as the analytic sample for outcome analyses. This flow documents reasons for exclusion (e.g., those who took the class previously were not part of the analytical survey) and those lost to follow-up at the end of the program.

A total of 234 participants were enrolled in the program. Among this initial group, 181 (77.4%) individuals were potentially eligible for the outcomes study, 21 (9.0%) individuals did not meet study criteria (e.g., to be considered active, participants needed to attend either the first or second training session), and 32 (13.7%) individuals were repeaters (previous *Fit & Strong!* participants). Among the 181 potentially eligible participants, however, only 136 (75.1%) completed the baseline survey and were, therefore, eligible to be part of the initial participant comparison analyses. Only 71 participants (39.2%) of the 136 eligible participants completed *both* pre and post-test surveys and served as “impact study participants.”

### Data analysis

Characteristics of those who completed both baseline and post-test surveys (matched surveys) were compared to the other participants (non-matched surveys; those who only completed a baseline survey) using χ^2^ tests for categorical variables and two-sample *t*-tests for continuous variables. Next, association between participant characteristics and program completion status for the analytical sample was identified using logistic regression with odd ratios. The impact of *Fit & Strong!* was then evaluated by comparing the outcome measures using various methods (paired-*t*-test for continuous scales, Wilcoxon Signed Rank Test for ordinal scales, and McNemar test for two-level categorical scales).

## Results

### Objective 1: Study characteristics

As shown in Table 1, the average age of eligible *Fit & Strong!* participants (including all enrollees with baseline surveys) was 73.02 (SD = 9.16) years (49.3% were age 75 and older, and 35.0% were between the ages of 65 and 74). The majority of participants were female (80.2%) and were of non-Hispanic White ethnicity (82.8%). Over 75% had more than a high school degree, and 62.2% were married. Among the four chronic conditions reported (diabetes, hypertension, heart disease, and respiratory problems), hypertension was most frequently reported among the participants (47.3%). Of the 136 eligible participants who completed the baseline survey, 33.8% were from rural counties and 66.2% were from an urban county.

**Table 1:** **Baseline characteristics of eligible participants by data availability (i.e., presence of both baseline and post-test surveys)**.

Baseline characteristics	Categories	Eligible participants with baseline survey (*n* = 136)^a^	Eligible participants excluded from the impact study (*n* = 65)^b^	Impact study participants (*n* = 71)^c^	*p*-value*
Age	<75	68 (50.7%)	29 (44.6%)	39 (56.5%)	0.168
	≥75	66 (49.3%)	36 (55.4%)	30 (43.5%)	
	Mean (SD)	73.02 (±9.16)	74.25 (±9.82)	71.87 (±8.39)	0.134
Gender	Male	26 (19.8%)	18 (27.7%)	8 (12.1%)	0.025
	Female	105 (80.2%)	47 (72.3%)	58 (87.9%)	
Race/ethnicity	White (not Hispanic origin)	106 (82.8%)	52 (85.2%)	54 (80.6%)	0.486
	Non-White	22 (17.2%)	9 (14.8%)	13 (19.4%)	
Education	≤High school graduate	30 (23.3%)	17 (26.6%)	13 (20.0%)	0.378
	>High school graduate	99 (76.7%)	47 (73.4%)	52 (80.0%)	
Marital status	Married	84 (62.2%)	38 (58.5%)	46 (65.7%)	0.385
	Not married	51 (37.8%)	27 (41.5%)	24 (34.3%)	
Site	Rural	46 (33.8%)	19 (29.2%)	27 (38.0%)	0.279
	Urban	90 (66.2%)	46 (70.8%)	44 (62.0%)	
Reported number of chronic conditions^d^	Mean (SD)^e^	0.83 (±0.82)	0.94 (±0.90)	0.73 (±0.74)	0.168
	Median	1	1	1	
	Mode	0	1	0	
Chronic conditions	Diabetes	18 (13.5%)	11 (17.5%)	7 (10.0%)	0.209
	Hypertension	61 (46.6%)	28 (44.4%)	33 (48.5%)	0.640
	Heart disease	23 (17.3%)	13 (20.6%)	10 (14.3%)	0.334
	Respiratory problems	11 (8.3%)	7 (11.1%)	4 (5.8%)	0.270
Self-efficacy	Mean (SD)^e^	6.95 (±2.44)	6.48 (±2.47)	7.39 (±2.34)	0.033
RAPA (aerobic capacity)	Mean (SD)^e^	3.86 (±1.11)	3.86 (±1.12)	3.85 (±1.11)	0.991
RAPA (strength/flexibility)	None	73 (60.3%)	35 (60.3%)	38 (60.3%)	0.395
	Either	33 (27.3%)	18 (31.0%)	15 (23.8%)	
	Both	15 (12.4%)	5 (8.6%)	10 (15.9%)	
	Strength	22 (18.0%)	8 (13.6%)	14 (22.2%)	0.214
	Flexibility	44 (35.8%)	22 (37.3%)	22 (34.4%)	0.736
Self-rated health	Mean (SD)^e^	3.30 (±0.82)	3.14 (±0.85)	3.45 (±0.78)	0.028
Joint pain	Mean (SD)^e^	4.06 (±3.60)	4.21 (±3.75)	3.90 (±3.48)	0.636
Joint stiffness	Mean (SD)^e^	2.49 (±1.78)	2.59 (±1.86)	2.40 (±1.71)	0.555
Level of energy	Mean (SD)^e^	2.13 (±0.99)	2.18 (±0.98)	2.07 (±1.00)	0.527
***Fit & Strong!* attendance**
Completion status	Completed	76 (55.9%)	21 (32.3%)	55 (77.5%)	0.000
	Not completed	60 (44.1%)	44 (67.7%)	16 (22.5%)	
Total number of classes attended (MAX = 24)	Mean (SD)^e^	15.96 (±7.16)	11.40 (±7.58)	20.14 (±3.04)	0.000

*^a^*n* = 136 With a slight variation for each variable*.

*^b^*n* = 65 With a slight variation for each variable*.

*^c^*n* = 71 With a slight variation for each variable*.

*^d^Chronic conditions: heart diseases, diabetes, hypertension, and respiratory problems*.

*^e^SD, standard deviation*.

*p-value for statistical analyses (i.e., χ^2^ or *t*-tests) for comparing the enrollees with only baseline surveys and the enrollees with both baseline and follow-up surveys. Eligibility criteria for the *baseline analysis*, or the initial participant comparison analyses, included: (1) attendance of the first or second class sessions, (2) first-time participants (no previous participation in a *Fit & Strong!* class); and (3) completion of a baseline survey. Eligibility criteria for the *impact study analysis* included: (1) fulfillment of the aforementioned baseline analysis criteria, and (2) completion of a post-test survey. Participants who fulfilled the baseline analysis criteria and did not complete a post-test survey were excluded from the impact study analysis.

Compared to eligible participants omitted from the impact study because of lack of matched data (completed baseline and post-tests) (*n* = 65), a significantly larger proportion of impact study participants (*n* = 71) were female (87.9 vs. 72.3%, *p* = 0.025). On average, SE at baseline for impact study participants (*p* = 0.033) was significantly higher relative to eligible participants omitted from the impact study; whereas, average self-rated health (*p* = 0.028) at baseline for participants included in the impact study was significantly higher than participants who were not included in the impact study. There were no significant differences by other socio-demographic characteristics and baseline levels of physical activity and illness symptomatology.

### Objective 2: Class completion

As shown in Table 1, the average number of classes attended for all eligible participants who completed baseline surveys was approximately 16 (SD = 7.16) out of 24. The program completion rate was 55.9% (i.e., attending 18 or more of the 24 sessions). Significant differences were observed when comparing the completion rate and the number of classes attended between the two groups of eligible participants (impact analysis participants vs. non-impact analysis participants). On average, participants in the impact analysis group attended more sessions (average number of sessions attended = 20.14 vs. 11.40, *p* < 0.001) and had higher completion rates (77.5 vs. 32.3%, *p* < 0.001).

As further seen in Table 2, there were a few variables that differentiated the participants who did and did not complete the program. Participants from the rural sites were less likely to complete 18 or more classes than the participants from urban sites (OR = 0.41, *p* = 0.015). Those without any chronic conditions were also more likely to complete the program (OR = 2.34, *p* = 0.022); for every increase in number of chronic conditions, the odds of completing the class drops by 46.4%. There were no significant differences by other socio-demographic characteristics or baseline levels of physical activity, general health status, or illness symptomatology.

**Table 2:** **Comparison of participant baseline characteristics by their program completion status (i.e., attended at least 18 out of 24 sessions)**.

	*n*^a^	Program completion	
		Odds ratio	*p*-value^b^
Age	134 (98.5%)	1.008	0.685
Sex (female)	131 (96.3%)	0.433	0.063
Race/ethnicity (non-Hispanic White)	128 (94.1%)	1.562	0.357
Education (>high school graduate)	129 (94.9%)	1.882	0.147
Marital status (married)	135 (99.3%)	1.089	0.812
Site (urban)	136 (100.0%)	0.407	0.015
Reported number of chronic conditions	131 (96.3%)		
Indicative/binary^c^ (≥1)		2.337	0.022
Count^d^		0.536	0.006
Baseline self-efficacy	129 (94.9%)	1.113	0.145
Baseline physical activity (aerobic capacity) (active)	118 (86.8%)	0.724	0.400
Baseline physical activity (strength/flexibility)	121 (89.0%)		0.111
None	73 (60.3%)	0.521	0.301
Either	33 (27.3%)	0.268	0.053
Both (ref)	15 (12.4%)		
Baseline self-rated health	134 (98.5%)	1.309	0.213
Baseline joint pain	124 (91.2%)	0.942	0.234
Baseline joint stiffness	128 (94.1%)	0.933	0.488
Baseline level of energy	129 (94.9%)	0.882	0.487

*^a^Number of cases included in the analysis (maximum possible *n* = 136)*.

*^b^*p*-value from bivariate logistic regression model*.

*^c^Reported number of chronic conditions (0 = no chronic conditions; 1 = at least one chronic condition)*.

*^d^Reported number of chronic conditions (count variable ranging from 0 to 4)*.

### Objective 3: Impact of *Fit & Strong!*

According to the results illustrated in Table 3, in terms of primary outcomes, there were significant improvements in participants’ SE for exercise (*p* = 0.020, *d* = 0.30) and aerobic capacity (*p* = 0.022, *d* = 0.34) from baseline to post-test. In terms of the magnitude of improvement at the individual level, there was an 8.1% improvement in SE for exercise and an 11.9% improvement in aerobic capacity. Furthermore, 54.8% of the sample reported an improvement in confidence to exercise and a 29.8% improvement in aerobic capacity. Additionally, there was a shift in the proportion of participants who met the Surgeon General’s recommended physical activity guidelines (31). At baseline, 38.7% of the participants were determined to be “active” according to the Surgeon General’s guidelines; whereas, by the end of the program, 59.4% of participants were determined to be “active.”

**Table 3:** **Baseline and post-test comparisons for assessing the impact of *Fit & Strong!* program**.

Outcome	*n*^a^	Mean (SD)^b^		*p*-value	Effect size^c^	Improvement (%)	Percentage of improved participants (%)
		Pretest	Post-test			
**Primary outcomes**
Self-efficacy for adherence to exercise	62 (87.3%)	7.40 (±2.28)	8.00 (±1.99)	0.020	0.30	8.1	54.8
Aerobic physical activity level^d^	57 (80.3%)	3.85 (±1.11)	4.31 (±1.02)	0.022	0.34	11.9	29.8
**Secondary outcomes**
Self-rated health^d^		3.45 (±0.78)	3.49 (±0.73)	0.491	0.08	1.2	11.6
Weight	62 (87.3%)	172.02 (±33.87)	168.66 (±36.64)	0.056	0.25	2.0	14.5
Joint pain	62 (87.3%)	3.89 (±3.50)	3.73 (±3.62)	0.714	0.05	4.1	35.5
Joint stiffness	63 (88.7%)	2.38 (±1.73)	1.94 (±1.48)	0.017	0.31	18.7	17.5
Level of energy	63 (88.7%)	2.06 (±0.98)	1.82 (±0.94)	0.010	0.33	11.9	27.0
Strength^e^	59 (83.1%)	14 (22.2%)^f^	34 (52.3%)^f^	0.002			33.9
Flexibility^e^	59 (83.1%)	22 (34.4%)^f^	42 (65.6%)^f^	0.001			35.6
Strength and flexibility^d^	58 (81.7%)	0.56 (±0.76)	1.19 (±0.85)	0.000	0.59		48.3

*^a^Number of cases included in the analysis (maximum possible *n* = 71)*.

*^b^Mean and standard deviation (SD), unless otherwise indicated*.

*^c^Cohen’s *d* [effect sizes of *d* ≈ 0.2 (small), *d* ≈ 0.5 (medium), and *d* ≥ 0.8 (large)]*.

*^d^Wilcoxon’s paired sign rank test was used*.

*^e^McNemar test was used (No *t*-statistic)*.

*^f^Frequency and valid percentages*.

In terms of secondary outcomes, there were significant changes observed for joint stiffness, level of energy, and amount and intensity of physical activities related to strength and flexibility (*p* < 0.05). The effect sizes for all secondary outcomes ranged from 0.05 to 0.59. The strongest effect sizes were observed for strength and flexibility scales (*d* = 0.59), then for the level of energy (*d* = 0.33), and then joint stiffness (*d* = 0.31). At the individual participant level, there was a 19.2% improvement in the degree of joint stiffness and an 11.7% improvement in the level of energy. Furthermore, 17.5% of the participants reported improvements in joint stiffness and 27.0% reported improvements in the level of energy. Over one-third of participants reported improvements in the degree of physical activities related to strength. 35.6% reported improvements in the degree of physical activities related to flexibility, and 48.3% reported improvements in the degree of physical activities related to both strength and flexibility.

## Discussion

As with many evidence-based programs, the randomized trials often use a higher level of interventionists to provide a best case scenario (32, 33). Alternatively, translated models frequently use lay-leaders to expand dissemination efforts while minimizing costs (34, 35). The same is true of the original *Fit & Strong!* program, which originally used PTs or CEIs as class instructors as a means of minimizing harm to participants (7, 17).

The current study examined a lay-leader model of the *Fit & Strong!* program adapted to overcome common challenges to program implementation such as instructor availability (15, 16). Consistent with other findings showing successful applicability of lay-leaders with a variety of physical activity programs in diverse settings (34, 36–38), we saw many positive outcomes and recommend the implementation of a lay-led model. Our program fidelity observations (data not reported here) indicated that group facilitators with more experience in evidence-based programing tended to adhere more closely to program guidelines than instructors with no or limited prior experience adhering to scripted programs.

Our study resonates with previous literature that shows the value of lay-led programs for seniors, especially those with arthritis, which was the original target group for *Fit & Strong!* classes. Cohen et al. (39) compared a lay-led arthritis self-management course and professional-led arthritis self-management course and identified no significant differences for participant outcomes by leader type (although, it should be noted that the courses compared differed slightly in course content). Similarly, Lorig et al. (40) compared a lay-led and a professional-led arthritis self-management course, and both courses showed a significant increase in participant knowledge. Participants in the professional-led courses showed a greater gain in knowledge than those in the lay-led courses; however, participants from the lay-led model showed greater improvement in relaxation practice and higher attendance rates (40). These studies utilizing lay-leaders for physical activity programs confirm the feasibility of using a lay-leader model for increasing the availability and adoption of the *Fit & Strong!* program.

The completion rate for those in the impact study (77.5%) was comparable with that found in other research studies using different time-bound evidence-based programs (41). It is not surprising that those in rural areas vs. those in more urban areas were less likely to complete the program given the previously documented challenges to bringing health services or health promotion programs to rural areas (42, 43). Additionally, the fact that those with one or more comorbidities were less likely to complete classes can be attributed to the challenges reported by those facing multiple chronic conditions (44); although, more research is needed to understand how different conditions might affect completion rates. These findings suggest that additional efforts are needed to attract and retain participants from rural areas and those with multiple chronic conditions.

The current study also examined the impact of lay-led *Fit & Strong!* classes on various outcome measures. Participants showed a significant improvement in their aerobic capacity, joint stiffness, level of energy/fatigue, and SE for exercise. Participants also reported greater participation in exercise types (flexibility, strength, or both) such that more individuals met the Surgeon General’s recommendations of including exercises targeting flexibility and strength training. These findings are consistent with those of Hughes and colleagues (7, 11), who reported *Fit & Strong!* participant improvement for exercise efficacy, exercise adherence, joint stiffness, physical functioning, and exercise capacity. Hughes reported a 15.6% reduction in participants’ stiffness scores at post-test (7), which is consistent with the 19.2% reduction in stiffness scores for participants in the current study. Other measure comparisons could not be made because the current study used different outcome measures than those used by Hughes.

### Study limitations

There are several limitations to this pilot study that should be noted but are acceptable considering this was the initial investigation of a translated intervention. A major limitation for the generalizability of study findings is the small sample size for the final impact study. Additionally, compared to the original *Fit & Strong!* studies (7, 11), there was a relatively high attrition rate (47.9%) from pre to post-test, as commonly found in more community-oriented exercise programs (45). As documented by local program administrators, this high attrition rate was attributed to “loose program adherence/commitment” as some participants preferred “dropping into classes” (i.e., attend at their leisure) as opposed to fully committing to the 8-week program. Others, especially in the rural areas, had limited transportation and, therefore, had difficulties with program attendance. In the current study, we assessed outcomes only for those with complete data, and thus were not able to assess whether those who lacked complete data might have biased study results. However, when we compared the baseline characteristics of the eligible participants included and excluded from the impact analysis, we only found a few significant differences between those two groups, indicating the potential similarity of the two groups.

This study only included a post-test that was administered during the last week of the program. No follow-up assessments were administered after the last session. The lack of follow-up measurements after the program limited our ability to observe any potential long-term effects of utilizing the lay-led *Fit & Strong!* program. However, this study enabled the primary question to be addressed regarding applicability to a broader population of older adults and also the potential value of a lay-led approach for this program in other communities.

Other study limitations can be attributed to program design and evaluation issues. The participants were self-selected into the program from different delivery sites, creating a potential self-selection, or delivery site bias. Also, participants included in the impact analysis had higher SE and self-rated health than those who were not included in the impact analysis, potentially influencing the program impact analysis. This is not surprising given the literature to date suggesting that older adults with better health are more likely to attend and complete a health promotion program (46–48). Such relationships pose a potential intervention bias, which must be considered when interpreting study results.

Finally, participants from this study differed somewhat from participants for which the program was originally intended. Older adults in various physical capacities, including those who were more sedentary or suffered from “achy joints” were recruited for this iteration. In contrast, participants in the original randomized control trials were originally selected based upon the presence of lower-extremity joint stiffness and pain associated with osteoarthritis and related symptomatology. Consequently, it is not possible to do a direct comparison with the earlier studies by Hughes and colleagues (7, 11, 17) since the extent to which participants in the current study had arthritis and specifically osteoarthritis is unknown. Thus, outcomes for arthritis-related symptomatology may have been attenuated in this more generalized study population.

### Program implications and future research directions

An important implication of the study is that the *Fit & Strong!* program may benefit the general older adult population and not just those with lower-extremity osteoarthritis. This may be because a substantial proportion of older adults experience some type of joint pain and/or stiffness, not just specifically in the lower-extremity (49) Furthermore, as indicated from program facilitators, *Fit & Strong!* can benefit sedentary older adults who want a beginning level and less intimidating means to start a physical activity regiment. Thus, this program has universal benefits.

Although it is impossible to draw a definitive conclusion, these findings strongly suggest that the *Fit & Strong!* program can be instructed by lay-leaders with standardized training and continued support from the developers and/or on-site Master Trainers. This is important because the training provides lay-leaders (i.e., those without formal exercise or professional training) with guided instruction and ongoing feedback related to program administration as well as proper techniques for exercise progression. These modifications are essential for conducting the program and are seen as critical in allowing the *Fit & Strong!* program to be disseminated more broadly as a lay-led model.

There is now a growing literature on factors affecting recruitment and strategies for boosting program retention (50). Given the reported attrition levels in attendance from entry into the study till class completion, efforts to retain participants from start to finish should focus on committing and motivating participants to fully complete the program. This is often accomplished during enrollment of participants or during the first session, or orientation, of the program (51). Furthermore, instructors should emphasize to participants early on the benefits gained from full participation and should strive to interact and engage participants during sessions and outside of class where necessary (e.g., follow-up phone calls if a participant misses a class).

This pilot study also highlights the need for additional research. Future research should compare lay-led and professional-led *Fit & Strong!* classes in terms of the magnitude of program impact and program fidelity. Also, lay-led *Fit & Strong!* classes should be evaluated/assessed in other settings to draw a more generalizable conclusion about the utility and effectiveness of varying levels of instructor expertise and training components.

## Conclusion

Overall, utilizing a lay-led model was successfully adapted from the original *Fit & Strong!* program that relied on professional and experienced leaders (PTs and CEIs). The lay-led *Fit & Strong!* model produced outcomes that are consistent with the previous findings from the original intervention. Specifically, the program showed improvement in participants’ SE for exercise, aerobic capacity, engagement in strength, and flexibility exercises, while increasing energy levels and decreasing joint stiffness. The magnitude of program attrition in community-based exercise programs can be large; hence, creative strategies are needed to boost participant retention throughout the entire intervention period.

## Conflict of Interest Statement

The authors declare that the research was conducted in the absence of any commercial or financial relationships that could be construed as a potential conflict of interest.

This paper is included in the Research Topic, “Evidence-Based Programming for Older Adults.” This Research Topic received partial funding from multiple government and private organizations/agencies; however, the views, findings, and conclusions in these articles are those of the authors and do not necessarily represent the official position of these organizations/agencies. All papers published in the Research Topic received peer review from members of the Frontiers in Public Health (Public Health Education and Promotion section) panel of Review Editors. Because this Research Topic represents work closely associated with a nationwide evidence-based movement in the US, many of the authors and/or Review Editors may have worked together previously in some fashion. Review Editors were purposively selected based on their expertise with evaluation and/or evidence-based programming for older adults. Review Editors were independent of named authors on any given article published in this volume.
